# Oral Etoposide for Relapsed or Refractory Ewing Sarcoma in Adolescent and Adult Patients

**DOI:** 10.1155/sarc/8247342

**Published:** 2024-12-13

**Authors:** Louise Kostos, Victoria Rayson, Jayesh Desai, Lisa Orme, Susie Bae, Anne Hamilton, Stephen J. Luen, Jeremy Lewin

**Affiliations:** ^1^Department of Medical Oncology, Peter MacCallum Cancer Centre, Melbourne, Victoria, Australia; ^2^Sir Peter MacCallum Department of Oncology, University of Melbourne, Melbourne, Victoria, Australia; ^3^Victorian Adolescent & Young Adult Cancer Service, Peter MacCallum Cancer Centre, Melbourne, Victoria, Australia

**Keywords:** etoposide, Ewing sarcoma, metronomic chemotherapy, relapse, topoisomerase II inhibitors

## Abstract

Prognosis remains poor for patients with relapsed or refractory Ewing sarcoma, with limited treatment options after first-line therapy. Oral etoposide has efficacy in the paediatric setting; however, data are limited in adults. A retrospective analysis was conducted on 33 patients with relapsed or refractory Ewing sarcoma who completed at least one cycle of oral etoposide at the Peter MacCallum Cancer Centre from 2005 to 2020. The median age at diagnosis and first relapse was 21 and 23 years, respectively. All patients had prior exposure to intravenous etoposide. Nine patients (27%) had stable disease for at least 6 months, and six patients (18%) had a partial response. The clinical benefit rate was 45%. The median PFS was 3.6 months (95% CI: 1.7–5.5), and OS was 8.5 months (95% CI: 4.1–13.0). Despite prior exposure, oral etoposide demonstrated antitumour activity and durable responses in the relapsed or refractory setting for adult patients with Ewing sarcoma.

## 1. Introduction

Ewing sarcoma is a small round-cell malignancy typically arising from bone or soft tissue, with most cases driven by a pathognomonic chromosomal translocation between EWSRI and FLI1. Though most frequently occurring in patients between 10 and 15 years of age, up to 30% of the cases occur in adults over the age of 20 [1]. Multiagent chemotherapy and multidisciplinary care have dramatically improved the outcomes for patients with newly diagnosed Ewing sarcoma, with contemporary series demonstrating that between 70% and 80% of those with localised disease are cured [2]. Unfortunately, despite aggressive primary therapy, a proportion of patients with localised disease and 60%–80% of the patients with primary disseminated disease experience a relapse [2, 3]. Outcomes for those patients remain poor with a 5-year survival of approximately 15%.

When recurrent or primary refractory Ewing sarcoma is identified, the optimal approach in management is unclear given the limited randomised data available to guide clinical decisions [4]. In the most recent randomisation of the rEECur trial, high-dose ifosfamide demonstrated superiority compared to topotecan/cyclophosphamide (TC) in terms of event-free survival (EFS) and overall survival (OS). Use of high-dose ifosfamide, however, remains limited by clinician preference and side-effect profile, with 26% of the patients discontinuing treatment due to treatment-related adverse events including encephalopathy and renal toxicity [5]. Tyrosine kinase inhibitors (TKIs) and immune checkpoint inhibitors have also been studied in early-phase trials given success in other tumour types, with occasional clinical benefit seen using cabozantinib or regorafenib [6–8].

Low-dose metronomic single-agent etoposide, which induces DNA damage by inhibiting topoisomerase II [9], has clinical activity in both soft tissue sarcomas and osteosarcoma [10, 11]; however, evidence in Ewing sarcoma is limited. Podda and colleagues conducted a retrospective review of 58 paediatric patients with relapsed or refractory Ewing sarcoma who received low-dose oral etoposide (40 mg/m^2^ daily for 21 days out of 28 days) between 1989 and 2012 and reported significant clinical activity, with a 24% objective response rate (ORR) and 22% stable disease (SD) rate after 2-3 cycles [12]. To our knowledge, no such data exist in the adult population, though etoposide has been included in oral metronomic combination regimens [13]. Oral etoposide monotherapy may be an appealing option in the palliative setting which balances its ease of administration, clinical activity, and low toxicity rate. Literature suggests that oral is equivalent to intravenous dosing in efficacy across several tumour types [14, 15].

In this study, we present the first retrospective analysis of real-world adolescent and young adult (AYA) and older patients with relapsed or refractory Ewing sarcoma treated with single-agent metronomic oral etoposide and investigate its antitumour activity.

## 2. Methods

We conducted a retrospective review of patients with recurrent or refractory Ewing sarcoma treated at the Peter MacCallum Cancer Centre (PMCC), a large, adult tertiary cancer referral centre in Melbourne, Australia, between January 2005 and December 2020. Ethics approval was provided by the Human Research Ethics Committee (HREC) at PMCC (Project ID QA73503/PMCC) and was conducted in accordance with the Declaration of Helsinki. Patients were identified from pharmacy dispensing data and patient information sourced from the Electronic Medical Record (EMR) and paper medical files.

### 2.1. Patient Selection

**Figure 1:**
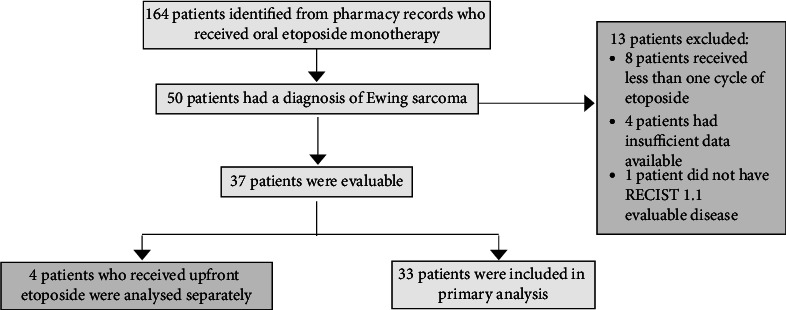
Trial schema.

Eligible patients had a diagnosis of relapsed or refractory Ewing sarcoma, had received oral etoposide monotherapy to manage disease recurrence or progression, and had evaluable disease according to RECIST 1.1 criteria. Out of 164 patients initially identified from pharmacy records, 50 patients had a diagnosis of Ewing sarcoma. A total of 33 patients were then included in the primary analysis (see Figure 1). We excluded patients who had incomplete data available and received less than one whole cycle of etoposide. All schedules of metronomic etoposide were included in the analysis. Patients' baseline and restaging CT and PET scan imaging were reviewed in addition to routine blood test results and medical documentation.

### 2.2. Evaluation Criteria

The primary objective of this study was to assess the clinical benefit rate (CBR), where CBR was defined as the percentage of patients who achieved SD that was sustained for at least 6 months, partial response (PR) or complete response (CR) according to RECIST 1.1. Secondary endpoints, all measured from the date of etoposide commencement, included progression-free survival (PFS), duration of response (DoR) and OS. Treatment tolerability was also evaluated by measuring the rate of treatment discontinuation and delays due to toxicity.

### 2.3. Statistical Analysis

IBM SPSS Statistics software was used to analyse the data. Descriptive statistics were used to describe baseline characteristics, percentages to represent categorical data and medians (ranges) for continuous data. Toxicity data were summarised using numerical counts. The Kaplan–Meier product-limit method was used to estimate OS and PFS for included patients. Survival was measured from the date of treatment commencement.

## 3. Results

### 3.1. Patient Characteristics

From 01 January 2005 to 01 December 2020, 33 patients were identified with relapsed or refractory Ewing sarcoma treated with single-agent oral etoposide and considered evaluable. Baseline patient details are listed in Table 1.

Twenty-three patients (70%) had the first disease relapse within 2 years of initial diagnosis, with the median DFI from diagnosis to first relapse being 20.8 months (range: 4.6–137.9). In terms of the first line of therapy, most patients (82%) received VDC-IE. All patients had previous exposure to intravenous etoposide during first-line or second-line chemotherapy regimens.

### 3.2. Treatment Disposition

On relapse, varying dosing regimens of oral etoposide were used, most commonly 100 mg daily for days 1–10 q21 (79%), followed by 100 mg daily for days 1–14 q21 (9%), and 100 mg/50 mg on alternating days on days 1–14 q21 (6%). The median number of cycles of oral etoposide given was 4.3 (range 1.0–46.3). Twelve patients (36%) received at least 6 months of treatment.

Twenty patients (61%) received further lines of treatment on progression. Most commonly, patients received irinotecan and temozolomide, single-agent cyclophosphamide, or topotecan and cyclophosphamide (see Table 2).

### 3.3. Efficacy

**Figure 2:**
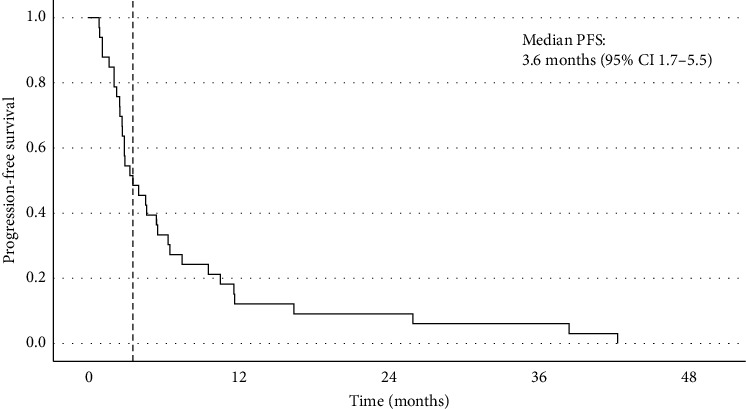
Progression-free survival.

**Figure 3:**
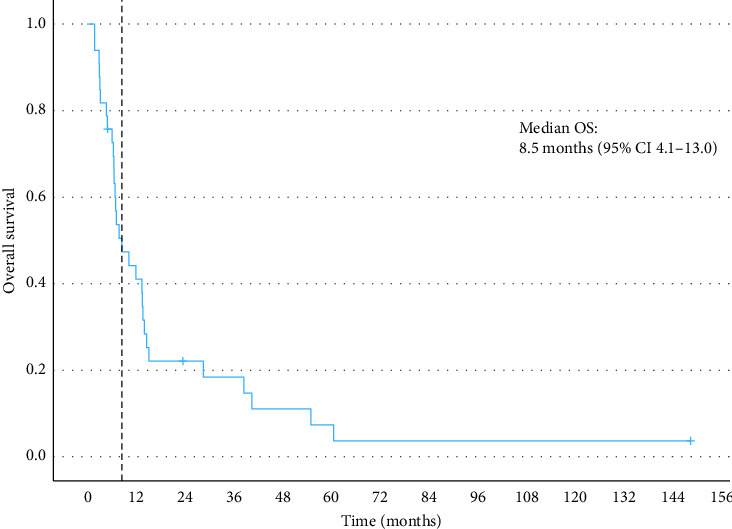
Overall survival.

**Figure 4:**
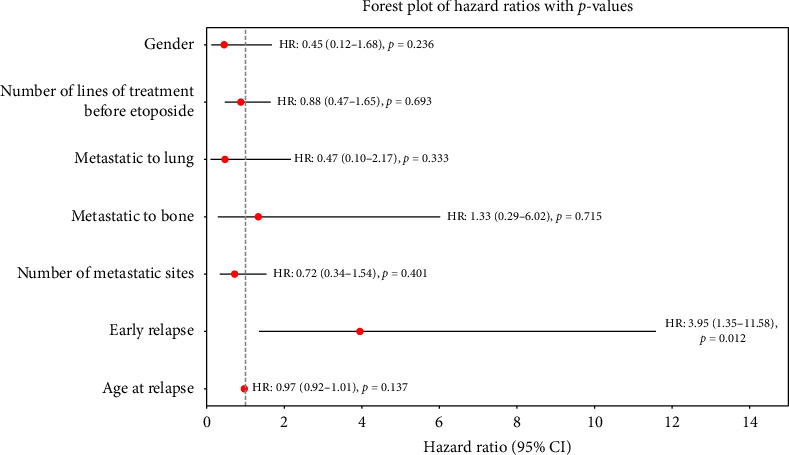
Forest plot of hazard ratios for overall survival. Early relapse is defined by time from initial diagnosis to first relapse less than 24 months.

In terms of best response according to RECIST 1.1, 39% of the patients had SD, 18% achieved a PR, and 42% had PD on the first assessment (see Table 3). There were no complete responses, and the ORR was 18%. Of the patients who achieved SD as best response, nine had this sustained for at least 6 months (27%). Including the patients with a PR and SD for at least 6 months, the 6-month CBR was 45%. For the patients who had SD or PR, the median duration of response (DoR) was 6.6 months. The median PFS was 3.6 months (95% CI: 1.7–5.5) (see Figure 2), and the median OS was 8.5 months (95% CI: 4.1–13.0) (see Figure 3). The median follow-up was 8.6 months. One patient who received oral etoposide for first relapse had a PFS of 42.5 months and an OS of 55.1 months. For those patients who relapsed within 2 years of diagnosis, the median PFS and OS were 2.9 months (95% CI: 2.6–3.2) and 7.0 months (95% CI: 6.1–7.9), respectively. On multivariable Cox regression analysis, early relapse was associated with a significantly increased risk of death (HR: 3.95 [95% CI: 1.35–11.58], *p*=0.012) (Figure 4). Those who had initial relapse after 2 years from diagnosis had longer OS and PFS, 6.4 (95% CI: 0.13–12.7) and 12.0 months (95% CI: 6.1–17.9), respectively.

### 3.4. Toxicity

Treatment was well tolerated, with only three (9%) patients requiring a dose reduction and eight (24%) requiring a dose delay. The most common reasons for a dose reduction included neutropaenia (*n* = 2), fatigue (*n* = 1) and infection (*n* = 1). The most common reasons for a dose delay were infection (*n* = 3) and neutropaenia (*n* = 3). Eight patients (24%) received concurrent radiotherapy; however, only one patient delayed etoposide due to this.

## 4. Discussion

Though a variety of chemotherapy regimens are available for patients with relapsed or refractory Ewing sarcoma, there is no standardised approach or sequence for clinicians to follow. Currently, the most active treatment with high-dose ifosfamide is occasionally limited by its toxicity profile and may require an inpatient stay to facilitate its administration. In an often-young patient population, it is imperative yet challenging to strike a balance between achieving disease response, whilst minimising toxicity and maintaining quality of life. The rEECur trial has provided some clarity as to potential treatment sequencing for patients with Ewing sarcoma, with high-dose ifosfamide more effective than TC, and the irinotecan and temolozomide, and gemcitabine and docetaxel arms being dropped early in the study as they were predicted to have low probability as a superior treatment, although this was not directly compared to high-dose ifosfamide [5]. As each salvage chemotherapy regimen carries different toxicity profiles, clinicians need to discuss the therapeutic landscape for relapsed Ewing sarcoma that balances the risk/benefit ratio, particularly for those wishing to maximize their quality of life.

Low-dose continuous oral etoposide offers a less-intensive and well-tolerated treatment alternative. In addition, it is economically appealing given the low cost, ready availability, and general ease of monitoring compared with other novel agents such as tyrosine kinase inhibitors. Etoposide targets topoisomerase II, thereby preventing the repair of double-stranded DNA breaks and leading to cell death. In contrast to high-dose cytotoxic chemotherapy, which targets the proliferating tumour cells themselves, low-dose metronomic chemotherapy targets the endothelial cells of the tumour vasculature, limiting their opportunity to repair DNA damage and thereby resulting in an antiangiogenic effect [16]. Preclinical data support low-dose oral etoposide, as the oral bioavailability decreases as the dose increases and intravenous doses are then required [17].

In terms of efficacy, our results are consistent with the paediatric study by Podda et al. [12] with an ORR of 18%. Perhaps, a more clinically meaningful measure is the CBR, which was 45% in our study, demonstrating that just below half the patients in this study had at least 6 months of nonprogression during treatment with oral etoposide. This was comparable to the CBR of 37% (17/46) in the study by Podda et al..

The median number of cycles was higher in our study compared with the younger population studied in Podda et al., (median of 4.3 vs. 3 cycles, respectively), which likely reflects the variation in etoposide dosing between the two studies. Treatment tolerability was challenging to further assess given the retrospective nature of this study, with the rate of grade 3 or higher haematological adverse events not captured in our study. Based on the small proportion of patients requiring a dose reduction (9%) or dose delay (24%), however, treatment appears to have been well tolerated in our cohort. Importantly, 61% of the patients in our cohort remained well enough to receive subsequent treatment after progression on oral etoposide.

As a retrospective cohort analysis of real-world patients, several limitations affect the quality of the data collected. First, unlike in a prospective registry, there was no control over the quality of the data collected. Four patients were excluded from the analysis due to insufficient data. We were limited in data capture for the 33 patients included in the study such as additional prognostic variables (including performance status and serum LDH) and toxicity enabling accurate grading. We excluded eight patients who did not receive one full cycle of oral etoposide. Six of these patients had rapid disease progression and were imminently dying at the commencement of etoposide, and two ceased treatment early due to presumed toxicity (lethargy and sepsis). In doing so, however, the survival outcomes in this analysis are presumably prolonged compared with if these patients were also included.

Furthermore, patients did not undergo imaging assessments at consistent time intervals which may falsely shorten or prolong the PFS. Data for OS are accurate due to hospital records confirming the start date of etoposide and the date of death. Only one patient was lost to follow-up and, therefore, was censored in the survival analyses. Finally, as this was a retrospective study, there was a lack of randomisation or the presence of a control arm. To determine the actual clinical benefit of oral etoposide, it should be compared with an existing standard therapy in a prospective study.

Importantly, given the lack of therapeutic options for patients with relapsed or refractory Ewing sarcoma, enrolment in clinical trials should always be considered if the patient is suitable.

## 5. Conclusion

Despite prior exposure, low-dose oral etoposide administered in a metronomic regimen demonstrated objective antitumour activity and durable responses for some AYA and adult patients with relapsed or refractory Ewing sarcoma in this retrospective study. Clinicians could consider this treatment option in their therapeutic arsenal, particularly given the acceptable toxicity and ease in administration.

## Figures and Tables

**Table 1:** Patient baseline characteristics.

Characteristic	*N* = 33 Number (%)
Gender	
Male	26 (79%)
Female	7 (21%)
Median age at diagnosis	21 years (range 15–53 years)
Stage at diagnosis	
Localised	26 (79%)
Metastatic	7 (21%)
Disease-free interval until first relapse	
< 2 years	23 (70%)
≥ 2 years	10 (30%)
Site of primary lesion	
Bone-axial	9 (27%)
Bone-extra-axial	16 (49%)
Soft tissue	8 (24%)
Location of metastatic lesions at relapse	
Pulmonary	27 (82%)
Bone	13 (39%)
Other	9 (27%)
Treatment of primary lesion	
Surgical resection alone	11 (33%)
Radiotherapy alone	9 (27%)
Both	12 (36%)
No treatment to primary	1 (3%)
Initial chemotherapy regimen	
VDC-IE	27 (82%)
VIDE/VAI	3 (9%)
Vincristine, ifosfamide and doxorubicin	2 (6%)
Vincristine, dactinomycin and ifosfamide	1 (3%)
Number of treatment lines prior to etoposide monotherapy (including primary treatment)	
1	10 (30%)
2	15 (46%)
≥ 3	8 (24%)

**Table 2:** Subsequent treatment/s following etoposide.

Subsequent treatment/s after oral etoposide	Number (%)
Received subsequent treatment line/s	20 (61%)
Did not receive subsequent treatment	13 (39%)
Number of further treatment lines received in relapsed setting (*n* = 20)	
1	17 (85%)
2	1 (5%)
3	1 (5%)
Unknown	1 (5%)
Subsequent treatments given	
Irinotecan/temozolomide	10
Cyclophosphamide	2
Topotecan/cyclophosphamide	3
Ifosfamide	1
Irinotecan	1
Etoposide (rechallenge)	1
Vincristine/doxorubicin/cyclophosphamide	1
Clinical trial	1
Liposomal doxorubicin/temsirolimus	1
Dacarbazine	1

**Table 3:** Best RECIST1.1 responses.

Response outcomes	Number (%)
SD	13 (39%)
SD for ≥ 6 months	9 (27%)
PR	6 (18%)
CR	0
PD	14 (42%)
ORR (PR + CR)	18%
CBR (SD for ≥ 6 months + PR + CR)	45%

## Data Availability

The dataset analysed during the current study is available from the corresponding author upon reasonable request.
